# Percentage of patients shifting to another treatment modality: An experience-guided decision

**DOI:** 10.1590/2177-6709.29.1.e2423133.oar

**Published:** 2024-03-29

**Authors:** Shivangi KUMARI, Manish GOYAL, Mukesh KUMAR, Mannu KHANNA, Ekta YADAV, Tanisha SINGH

**Affiliations:** 1 Teerthanker Mahaveer Dental College, Department of Orthodontics and Dentofacial Orthopaedics (Moradabad/Uttar Pradesh, India).

**Keywords:** Experience-based patients’ opinion, Visual Analogue Scale, Orthodontic treatment modality, Convert to alternative therapy, Opinião dos pacientes baseada na experiência, Escala visual analógica, Modalidade de tratamento ortodôntico, Conversão para terapia alternativa

## Abstract

**Objective:**

This study aimed to assess the frequency with which orthodontic patients decided to shift to another type of orthodontic appliance, among conventional metal brackets, ceramic brackets, lingual brackets and clear aligner, based on their personal experiences of pain, ulcers, bad breath, hygiene issues and social difficulties.

**Material and Methods:**

This study comprises of patients seeking orthodontic treatment. The sample (n = 500; age group = 19-25 years) was divided equally into four groups based on the treatment modality: conventional metal brackets, ceramic brackets, lingual brackets and clear aligner. Patients rated the questionnaire using a visual analogue scale, to assess variables (such as pain, ulcer etc) that impact various treatment modalities. Subsequently, patients from all groups provided feedback regarding their treatment experiences, and expressed their preference for an alternative modality. Intergroup comparison among the four groups was done using one-way analysis of variance with Tukey’s HSD *post-hoc* test (*p* ≤ 0.05).

**Results:**

Patients who received lingual brackets reported higher levels of pain and ulceration, as compared to those who received clear aligners. All four groups showed statistically significant differences for ulcers during treatment (*p* ≤ 0.05). Of the 125 patients who received conventional metal brackets, 28% expressed a preference for clear aligner therapy, while 20% preferred ceramic brackets. In the lingual group, 56% of 125 patients preferred clear aligner therapy, and 8% preferred ceramic brackets to complete their treatment. In the ceramic group, 83% did not want to switch, whereas 17% desired to switch to clear aligner, while in aligner group no patient desired to switch.

**Conclusions:**

A higher percentage of patients from lingual brackets group chose to shift to clear aligners, followed by conventional metal brackets group and by ceramic brackets group, in this descending order. The clear aligner group demonstrated fewer issues than the other treatment modalities.

## RESUMO

**Objetivo:**

Este estudo teve como objetivo avaliar a frequência com que pacientes ortodônticos decidiram mudar para outro tipo de aparelho ortodôntico, entre braquetes convencionais de metal, braquetes cerâmicos, braquetes linguais e alinhadores transparentes, com base em suas experiências pessoais de dor, aftas, mau hálito, problemas de higiene e dificuldades sociais.

**Material e Métodos:**

Esse estudo foi composto por pacientes que procuram tratamento ortodôntico. A amostra (n = 500; faixa etária = 19-25 anos) foi dividida igualmente em quatro grupos, com base na modalidade de tratamento: braquetes metálicos convencionais, braquetes cerâmicos, braquetes linguais e alinhadores transparentes. Os pacientes responderam a um questionário, usando uma escala visual analógica, para avaliar variáveis como dor e aftas, que impactam diferentes modalidades de tratamento. Posteriormente, os pacientes de todos os grupos forneceram *feedback* sobre suas experiências de tratamento e expressaram sua preferência por uma modalidade alternativa. A comparação intergrupos entre os quatro grupos foi feita usando análise de variância unidirecional com teste *post-hoc* HSD de Tukey (p ≤ 0,05).

**Resultados:**

Os pacientes que usaram braquetes linguais relataram níveis mais elevados de dor e aftas, em comparação com aqueles que usaram alinhadores transparentes. Todos os quatro grupos apresentaram diferenças estatisticamente significativas para aftas durante o tratamento (p ≤ 0,05). Dos 125 pacientes que usaram braquetes metálicos convencionais, 28% expressaram preferência pelo tratamento com alinhadores transparentes, enquanto 20% preferiram braquetes cerâmicos. No grupo com braquetes linguais, 56% dos 125 pacientes preferiram o tratamento com alinhadores transparentes e 8% preferiram braquetes cerâmicos para completar o tratamento. No grupo com braquetes cerâmicos, 83% não queriam trocar de tratamento, enquanto 17% desejavam mudar para os alinhadores transparentes; enquanto no grupo de alinhadores nenhum paciente desejou mudar.

**Conclusões:**

Uma porcentagem maior de pacientes do grupo com braquetes linguais optou pela mudança para alinhadores transparentes, seguido pelo grupo com braquetes metálicos convencionais e pelo grupo com braquetes cerâmicos, em ordem decrescente. O grupo de alinhadores transparentes demonstrou menos problemas do que as outras modalidades de tratamento.

## INTRODUCTION

Orthodontic advancements, particularly in recent years, have been followed by a large increase in patients’ aesthetic and comfort demands. Most studies in this field concentrated solely on the pain experienced by orthodontic patients during treatment. Orthodontic patients are often warned that there may be some discomfort related with the insertion of separators and initial archwires, as well as during periodic orthodontic appliance adjustments. The severity and length of discomfort, on the other hand, are not always discussed. Orthodontic treatment has a variety of negative effects that must be considered while comparing treatment options. In light of the healthcare industry’s current emphasis on patient demands, it is important to evaluate patients’ experiences throughout treatment.

Although orthodontic therapy has been shown to be effective for achieving occlusal functions and aesthetics demands, many individuals are hesitant to seek treatment, due to the discomfort associated with metal brackets.^1^ Using conventional metal brackets to align teeth often results in an unusual aesthetic that some people describe as “metallic mouth” or “train tracks.” In the late 1970s, patients were given an exciting new option for improving their aesthetics, with the introduction of lingual brackets. Adults who are self-conscious about their appearance typically wear them because they are hidden behind the teeth.^2^ Another great characteristic of lingual brackets is that they are less likely than traditional metal brackets to cause white spot lesions, but functional difficulties and a prolonged adaptation reduced its use until recent years.^3^ ^,^ ^4^ The most significant drawbacks were the increased price of laboratory equipment and the technical restrictions imposed on the staff.^5^ ^,^ ^6^ Previous studies examining patient satisfaction with fixed appliances have suggested that some patients may experience pain and discomfort immediately after orthodontic brackets are bonded, as well as sometimes after regular visits. This was negatively correlated with patient satisfaction.^7^

The introduction of clear aligners has drastically changed the face of cosmetic dentistry. Since their introduction to the market in 1997 by Align Technologies™, clear aligners have quickly become one of the most preferred orthodontic appliances for patients who are concerned with aesthetics.^8^Clear aligner system is one of the most sought by patients because of their aesthetics and comfort, compared with other types of treatments. Nevertheless, this system presents limitations in relation to correction of malocclusion, because aligners are deficient in some aspects, such as increase of teeth inclination after use; lack of control of tooth movement, which reflects in their deficiency to rotate roots, considering that in these cases there is need for overcorrection, interproximal accessories or reduction; in addition to having little successful in promoting dental occlusion and performing intrusion and extrusion of teeth. Also, their use is dependent on the cooperation of patients in using them for the recommended period.^9^

Orthodontists have long acknowledged that malocclusion and dentofacial anomalies can cause significant physical, social, and psychological distress.^10^ ^,^ ^11^ In order to assess orthodontic need and determine the effects of orthodontic care, patient-centred assessments are increasingly being used.^12^ ^,^ ^13^ There is some awareness of what patients hope to achieve with orthodontic treatments, and there is mounting proof that their positive opinions of the procedure have helped them.^14^ ^,^ ^15^ There is, however, limited knowledge of what patients anticipate from the course of orthodontic therapy and from potential side effects beyond discomfort. 

Thus, the goal of this study was to determine which group of patients experienced the most discomfort and other related issues, along with the percentage of adult orthodontic patients who would like to convert to another treatment modality after learning about the disadvantages of various treatment modalities. Conventional metal brackets, ceramic brackets, lingual brackets, and clear aligners were evaluated. 

## MATERIAL AND METHODS

Approval from the Institutional Ethical Committee of Teerthanker Mahaveer Dental College was obtained for the study (IEC/21-22/OD04). The prospective study was planned to determine patients’ experience undergoing treatment with conventional metal orthodontic brackets (Ormco, 3M Unitek), ceramic brackets (3M Unitek), lingual brackets (Incognito, 3 M Unitek, Monrovia) and clear aligner therapy (Aligner, Mumbai, India) after the first six to nine weeks of orthodontic treatment. The patients who came to the Department of Orthodontics and Dentofacial Orthopaedics at the aforementioned institution for their treatment from January 2022 to February 2023, aged between 18-25 years irrespective of their gender, were included in this study. The interval for changing the aligners was 20 days and for changing the orthodontic archwire was 28 days, so that all four groups had one appointment per month.

Based on the 95% power of the study and 5% type I error and effect size of 0.18, the minimum sample size was calculated to be 530 patients (conventional metal orthodontic brackets, ceramic brackets, lingual brackets and clear aligner therapy). The formula used for this was:

$$\eta_{A}=\left(\sigma_{A}^{2}+\sigma_{B}^{2}/\kappa \right){\left(\frac{Z_{1-\alpha }/\tau +Z_{1-\beta }}{\mu_{A}-\mu_{B}}\right)}^{2}$$

$$\eta_{B}=K\eta A$$

$$1-\beta =\phi \left(\frac{\mu_{A}-\mu_{B}\sqrt{\eta A}}{\left(\sigma_{A}^{2}+\sigma_{B}^{2}/\kappa \right)}-Z_{1-}\alpha /\tau \right)$$

Where-

$\kappa =\eta_{A}/\eta_{B}$ is the matching ratio

𝜎 is standard deviation

𝜎_A_ is standard deviation in Group “A”

𝜎_B_ is standard deviation in Group “B”

Φ is the standard Normal distribution function

Φ^-1^ is the standard Normal quantile function

𝛼 is Type I error

𝜏 is the number of comparisons to be made

𝛽 is type II error, meaning 1 - 𝛽 is power

The exclusion criteria were: severe crowding (> 8 mm) or extractions, history of orthodontic treatment, use of auxiliaries during or before the study period (e.g., expanders, transpalatal arch, temporary anchorage devices), oral pathology, a significant medical history or medication usage, patients younger than 18 years of age. The responses to the questionnaire having 20% or more of missing data were also excluded. 

Five hundred patients who were undergoing orthodontic treatment with conventional metal brackets, ceramic brackets, lingual brackets, or aligners were selected for this study. The treatment modality was chosen by the patient based on their personal preferences and financial circumstances. From the total sample, 94.3% responded to the questionnaire, allowing for dividing the final sample evenly between patients who used conventional metal brackets (n=125), ceramic brackets (n=125), lingual brackets (n=125) or clear aligners (n=125). A total of 0.7% of the patients did not respond to the questions due to their lack of interest in participating in the study.

**Figure 1:**
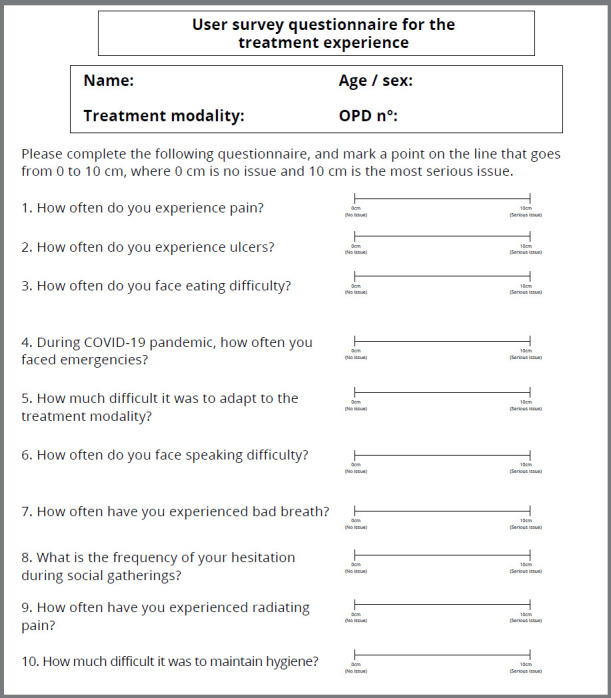
User survey questionnaire for the treatment experience.

After providing a detailed information about the survey, an informed consent was taken from the patients. A questionnaire was used to measure patients’ expectations of orthodontic treatment after 6-9 weeks (Fig 1). For this, a visual analogue scale (VAS) was used, with a line going from 0 to 10 cm, where 0 cm is no issue and 10 cm is the most serious issue. Patients were invited to answer a questionnaire about problems like pain, ulcers, bad breath (Fig 1), hygiene issues, hesitation while smiling in social gatherings, difficulties with speech, eating limitations, Covid-19 emergencies, and difficulties in adaptation. Initially, a pilot study was conducted to estimate the time required to complete the questionnaire, which confirmed that the time required to complete the questionnaire was 1 hour. After that, the patients from each group were interviewed together by the same interviewer and given the option to express their views regarding the pros and cons of the different treatment modalities. They were interviewed in the same environment, so that they could listen to the experience of the other patients and have a better understanding of the options available to them, thus making more informed decisions.

Following the discussion, patients were asked if they wished to switch to an alternative type of brackets. An audio recording of the interview was conducted, with the purpose of emphasizing the content and verbal prompts, facilitating the interviewer in producing a “verbatim transcript” of the interview.^16^

The response from each patient was measured on the visual analogue scale with a vernier caliper. To ensure consistency, a subset of data was measured again after a two-week period. The readings were rounded off to the nearest centimeter. 

## STATISTICAL ANALYSIS

Descriptive and inferential statistics were analyzed using IBM SPSS v. 20.0 (IBM Corp. Released 2011. IBM SPSS Statistics for Windows, Armonk, NY: IBM Corp). Mean and standard deviation were used to summarize the responses given by the use of VAS. The data normality was checked using the Kolmogorov-Smirnov test, and the distribution was found to be normal. The sample’s homogeneity was confirmed by Levene’s test and the intraobserver reliability was measured after two weeks, for the evaluation of intraclass correlation coefficient. Intergroup comparison among the four groups was done using one-way analysis of variance with Tukey’s HSD *post-hoc* test. Throughout the study, *p*-values <0.05 were considered as statistically significant. 

## RESULTS

The intraclass correlation values ranged from 0.788 to 0.988, showing good intraobserver agreement.

The pain and ulcer were most commonly experienced by the patients who were undergoing treatment with lingual brackets (9.62±0.49, 9.56±0.64), followed by metal brackets (8.60±0.70, 8.95±1.02), ceramic brackets (7.34±1.6, 8.07±1.07) and clear aligners (1.98±0.06, 1.48±0.58), respectively. Patients with lingual brackets complained about ulcers on tongue, while patients with conventional metal brackets complained of ulcers on buccal mucosa. For pain, all the groups showed significant difference among each other, except for the metal brackets and lingual brackets. However, for ulcer, all four groups showed significant difference among each other (*p*<0.05) (Table 1). Regarding emergencies during Covid-19, patients using metal brackets (9.07±0.60) and lingual brackets (9.05±0.65) reported more orthodontic emergencies, followed by ceramic brackets (8.73±1.10) and clear aligner (1.15±0.38). A significant difference was seen between all groups, except for the metallic and ceramic brackets (*p*<0.05) (Table 1).

**Table 1:** Comparison of groups regarding pain, ulcer, emergency during Covid-19, eating difficulty, adaptation difficulty, hesitation, speech, food impaction, and bad breath.

Variables	Metal brackets	Ceramic brackets	Lingual brackets	Clear aligner	*p-* **value**
Pain	8.60±0.70^a^	7.34±1.64^b^	9.62±0.49^a^	1.98±0.60^c^	0.001*
Ulcer	8.95±1.02^a^	8.07±1.07^b^	9.56±0.64^c^	1.48±0.58^d^	0.001*
Emergency during Covid-19	9.07±0.60^a^	8.73±1.10^a^	9.05±0.65^b^	1.15±0.38^c^	0.001*
Eating difficulty	8.74±0.97^a^	8.75±0.94^ab^	8.57±0.85^b^	1.97±0.62^c^	0.001*
Adaptation difficulty	7.42±1.32^a^	7.43±1.70^ab^	8.46±1.02^b^	1.65±0.55^c^	0.001*
Hesitation	9.18±1.14^a^	4.22±1.11^b^	1.72±1.08^ce^	1.23±0.44^de^	0.001*
Speech	6.52±0.92^a^	4.96±1.53^b^	9.16±0.96^c^	1.88±0.54^d^	0.001*
Food impaction	8.83±1.13^a^	7.89±1.17^be^	7.76±2.50^ce^	1.31±0.48^d^	0.001*
Bad breath	8.02±1.71^a^	5.97±0.99^ad^	4.75±2.24^bd^	1.41±0.49^c^	0.001*

The statistical significance in the same row is shown by different superscript letters between different groups (HSD Tukey’s *post-hoc* test). *Statistically significant difference among groups.

The aligner patients had the least difficulty in eating (1.97±0.62) and adaptation (1.65±0.55), among all groups. Discomfort after the first- and second-week adjustments was also consistently less for aligner than other groups. For both adaptation and eating difficulty, the conventional metal brackets group showed significant difference with lingual brackets and clear aligner. However, the ceramic and lingual brackets groups were significantly different with the clear aligner group (*p*<0.05). The conventional metal brackets (9.18±1.14) showed maximum hesitation among all groups, followed by ceramic brackets (4.22±1.11), whereas the patients using lingual brackets (9.16±0.96) had the maximum difficulty in speech. For both speech (1.88±0.54) and hesitation (1.23±0.44), aligner group showed least problem. All groups were significantly different for speech. 

The problems of food impaction and bad breath were maximum in conventional metal brackets (8.83±1.13, 8.02±1.71), followed by ceramic brackets (7.89±1.17, 5.97±0.99), lingual brackets (7.76±2.50, 4.75±2.24) and clear aligners (1.13±0.48, 1.41±0.49) respectively. Patients treated with conventional metal brackets reported brushing their teeth frequently, as compared with the clear aligner group. They also reported gingival irritation and inflammation. In terms of food impaction, all groups showed significant difference, except for ceramic and lingual brackets. For bad breath, conventional metal brackets, lingual brackets and clear aligner were found to be significantly different (*p*<0.05) (Table 1).

**Figure 2:**
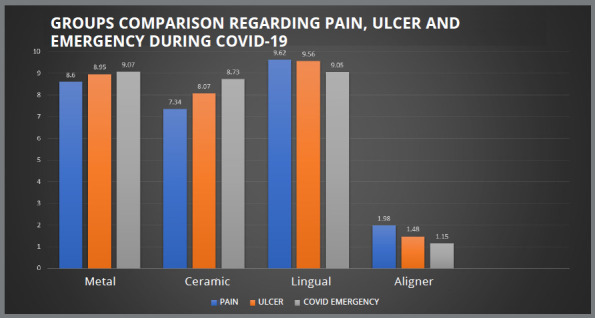
Groups comparison regarding pain, ulcer and emergency during Covid-19.

**Figure 3:**
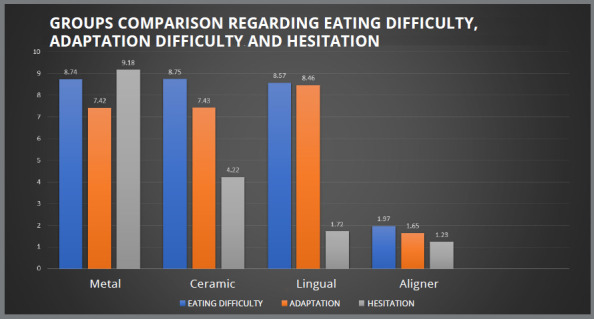
Groups comparison regarding eating difficulty, adaptation difficulty and hesitation.

**Figure 4:**
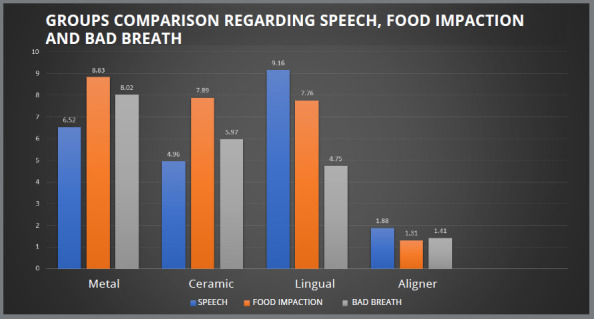
Groups comparison regarding speech, food impaction and bad breath.

Hesitation in social gatherings was found to have the greatest impact on the conventional metal brackets group, among all variables (9.18±1.14), and least in clear aligner group (1.23±0.44). Except for the clear aligner group, where there was no dietary restriction, patients of all three groups reported having trouble eating. Figures 2, 3 and 4 depict the comparison between the four groups for pain, ulcer, emergency during COVID-19 pandemic, eating difficulty, adaptation difficulty, hesitation, speech, food impaction, and bad breath.

**Figure 5:**
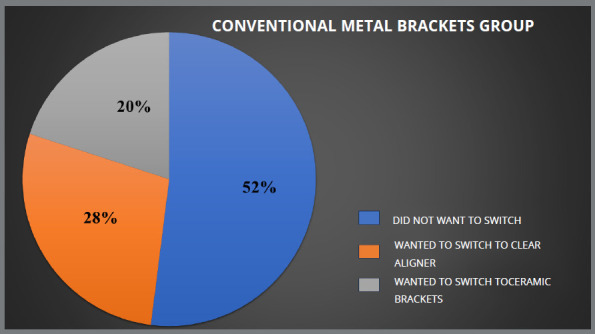
Percentage of patients in conventional metal brackets group who wanted to switch their treatment.

**Figure 6:**
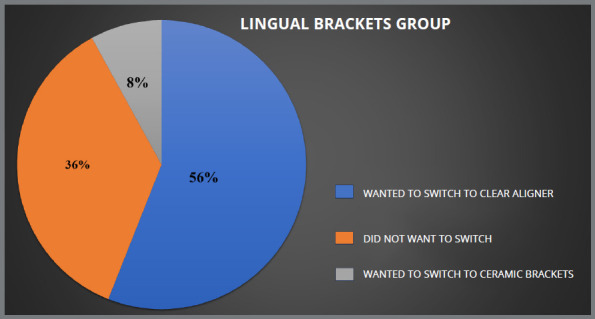
Percentage of patients in lingual brackets group who wanted to switch their treatment.

**Figure 7:**
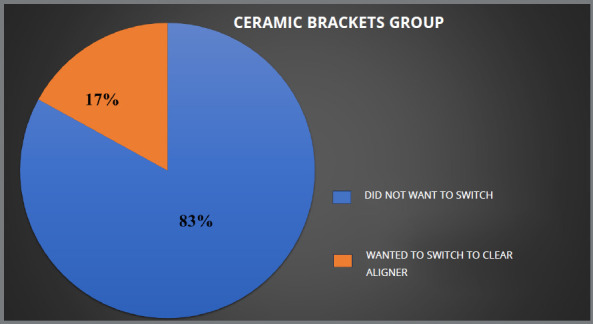
Percentage of patients in ceramic brackets group who wanted to switch their treatment.

**Figure 8:**
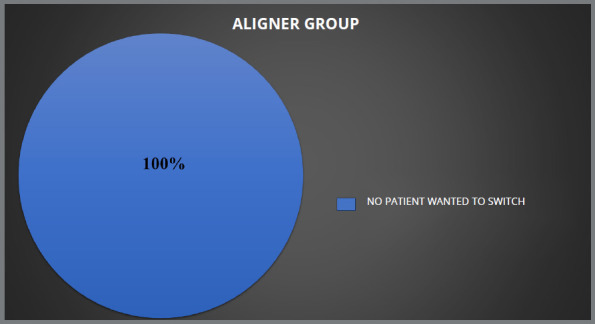
Percentage of patients in clear aligner group who wanted to switch their treatment.

Due to shared experiences and similar discomfort, patients in all groups (traditional metal brackets, lingual brackets, ceramic brackets and aligners) were open to switch to other appliance type. Those who opted for ceramic or lingual brackets reported roughly identical levels of satisfaction. Among the patients treated with conventional metal brackets, 28% were open to switch to clear aligner therapy and 20% wanted to switch to ceramic brackets, while the remaining were satisfied with the treatment modality they were undergoing (Fig 5). In the lingual brackets group, 56% expressed interest in switching to clear aligner therapy, 36% did not want to switch to another orthodontic appliance and 8% showed interest in switching to ceramic brackets (Fig 6). In the ceramic brackets group, 83% did not want to switch to another orthodontic appliance and the remaining 17% desired to change to the clear aligner group (Fig 7). In aligner group, no patient wants to switch to other treatment modality (Fig 8).

## DISCUSSION

It is highly recognized that the patients’ perceptions of treatment experiences and outcomes from orthodontic therapy are critical factors to consider when developing evidence-based orthodontic practice. 

The pain and discomfort are common side effects of orthodontic treatment.^16^ ^-^ ^18^ Fear of pain is one of the main reasons why patients don’t seek orthodontic care.^19^ Miller et al.^20^ discovered that patients with conventional buccal brackets experienced more severe pain than patients with lingual brackets. However, in this study, the patients with lingual brackets experienced more pain than other orthodontic therapy, which was consistent with a study done by Shalish et al.^21^

Wu et al.^22^ reported higher analgesic usage in lingual brackets patients, despite no statistically significant changes in pain levels between buccal and lingual brackets patients, which was similar to the finding of the present study.

Statistically significant differences were observed between the groups with respect to eating disturbances. Previous studies have reported that individuals with lingual impairments exhibited a greater incidence of eating disturbances and an extensive recovery period^7^, which was not consistent with this study, in which the aligner treatment exhibited the least degree of impairment and eating disturbances. Clear aligner patients experienced less impact on their oral health and daily routines than those with lingual, buccal, or ceramic brackets.^20^

The perception of orthodontic treatment among adult seeking care and the subsequent recommendation of orthodontic therapy for these patients are significantly influenced by psychological adaptation or a sense of well-being. This aspect of orthodontic care and its quantification assumes greater significance in the era of social media, in which opinions and experiences (both positive and negative) are eagerly accessible.^23^ Similarly, this study demonstrated that patients using conventional metal brackets were more conscious about their smile and looks in social gatherings. 

The patients using clear aligner appliances noticed discoloration, but not from the clear aligner itself, but from attachments. This issue does not exist in the other metal and lingual orthodontic treatment modalities. Patients in the ceramic group frequently complained about staining of their brackets. Although ceramic brackets are more aesthetically pleasing than metal brackets, polycrystalline brackets stain. This is most likely due to the individual’s diet, which includes a high intake of caffeine and colas.^25^

The pandemic has had a continuing effect on the orthodontic treatment, as the orthodontic treatment is a long process that requires regular visits.^26^ According to Bilder et al^27^, an orthodontic emergency may arise or quickly worsen, necessitating immediate action. Additionally, emergencies may result in oral mucosa or gingiva infection or excruciating pain, according to literature searches performed in PubMed and Embase databases.^27^ In this study, it was observed that a higher frequency of emergencies was associated with conventional metal and lingual brackets as opposed to clear aligners. During the ongoing months of the Covid-19 pandemic, a growing interest in the Invisalign method has been noted. When comparing the treatment duration and chair time, aligners have significant advantages.^26^ When compared with fixed appliances, the clear aligners have better aesthetic and oral hygiene, and are less affected by orthodontic emergencies and skipped visits, since the patient is provided with a set of aligners; hence patients tend to prefer clear aligners over conventional brackets.^28^

In this study, 48% of patients in the conventional metal brackets group, 62% in the lingual brackets group, and 17% of patients in ceramic brackets group wanted to switch to another treatment modality, while no patient in the aligner group wanted to switch treatment. Hohoff et al^29^ found that women under the age of 40 preferred lingual brackets over buccal brackets, while Nedwed and Meithke’s^30^ showed that women aged 20 to 30 were more likely to choose Invisalign^TM^ over buccal or lingual brackets. In this study, aesthetics (in comparison to buccal brackets), pain, and ulcers (lingual brackets) influenced patients’ choices, while a study by Kravitz et al.^31^ showed that one in every six patients (17.2%) needed to switch from Invisalign to brackets to finish their treatment. The difference between the two studies may be because this study was based solely on personal experience, and treatment was ongoing, whereas in the study by Kravitz et al. ^31^ patients had to switch because treatment could not be completed by one modality.

To our knowledge, no previous study has been done regarding patient experiences and their preference to switch treatment. In this study it was found that 28% of the patients in the conventional metal brackets group wanted to switch to clear aligner. On the other hand, 20% of the participants in the same group wanted to switch to ceramic brackets, while the remaining were satisfied with the treatment modality that they were undergoing. In the lingual brackets group, 56% of the participants wanted to switch to clear aligner therapy, and 8% wanted to switch to ceramic brackets, while the remaining participants preferred to continue with their ongoing treatment. Although the majority of published research on clear aligners has focused on its objective results, it may be useful to also assess how patients feel about it, in comparison to conventional fixed orthodontic appliances. Since patients’ opinions on which appliance is best for them can influence clinicians’ recommendations, comparing the available options is crucial.

Clinicians must carefully develop a suitable therapeutic approach based on current scientific evidence in order to successfully administer orthodontic treatment. Even though this is not the only thing that makes the final decision, clinical experience and the patient’s view are also important. 

## CONCLUSIONS

1. Patients using lingual brackets experienced more pain and ulcers, while the clear aligner group was the least affected. All groups were statistically significant in terms of having ulcer during the treatment (*p*<0.05). Patients using conventional metal brackets and lingual brackets had more orthodontic emergencies during Covid-19 pandemic.
2. Patients using conventional metal brackets had the worst breath and maximum food impaction. Both conventional metal brackets and lingual brackets patients wanted to switch their treatment to other treatment modality.
3. Social hesitation was found to have the most significant effect on the conventional metal brackets group, compared to other factors. The ceramic brackets patients experienced a notable degree of eating difficulty. The groups using lingual brackets and clear aligners exhibited greater pain levels, relative to other variables.
4. In the ceramic brackets group, 83% did not want to switch to another orthodontic appliance, and the remaining 17% desired to move to the clear aligner group.
5. In the lingual brackets group, 36% did not want to switch to another orthodontic appliance, 56% expressed interest in switching to clear aligner therapy, and 8% showed their interest in switching to ceramic brackets.
6. Among the patients treated with conventional metal brackets, 52% did not want to switch to another orthodontic appliance, 28% wanted to switch to clear aligner therapy, and 20% wanted to switch to ceramic brackets.
7. Within the aligner group, no patient expressed desire to switch to another treatment modality.

There are other factors that may impact the preference of a patient for different types of orthodontic treatments, such as the aesthetic impact of the appliance, as well as other factors that were highlighted in this study. Listening to the patients’ opinions, rather than simply reading about their experiences, can provide greater clarity for other patients when making decisions and choosing the best option for themselves.

## LIMITATIONS

The findings of the present study are only applicable to patients with minor malocclusion and for the initial length of time investigated in this study. Future randomized clinical trial are needed, with more complex malocclusion and for longer period of time. 
